# A novel gene-expression-signature-based model for prediction of response to Tripterysium glycosides tablet for rheumatoid arthritis patients

**DOI:** 10.1186/s12967-018-1549-9

**Published:** 2018-07-04

**Authors:** Yanqiong Zhang, Hailong Wang, Xia Mao, Qiuyan Guo, Weijie Li, Xiaoyue Wang, Guangyao Li, Na Lin

**Affiliations:** 1 Institute of Chinese Materia Medica, China Academy of Chinese Medical Sciences, Beijing, 100700 China; 2 Division of Rheumatology, Guang An Men Hospital, China Academy of Chinese Medical Science, Beijing, 100053 China; 3 Guiyang University of Chinese Medicine, Guiyang, 550025 China

**Keywords:** Personalized medicine, Rheumatoid arthritis, Tripterysium glycosides tablets, Gene expression profile, Gene signal transduction network, Partial least squares model

## Abstract

**Background:**

Approximately 30% of rheumatoid arthritis (RA) patients treated with Tripterysium glycosides (TG) tablets fail to achieve clinical improvement, implying the essentiality of predictive biomarkers and tools. Herein, we aimed to identify possible biomarkers predictive of therapeutic effects of TG tablets in RA.

**Methods:**

Gene expression profile in peripheral blood mononuclear cells obtained from a discovery cohort treated with TG tablets was detected by Affymetrix EG1.0 arrays. Then, a list of candidate gene biomarkers of response to TG tablets were identified by integrating differential expression data analysis and gene signal transduction network analysis. After that, a partial-least-squares (PLS) model based on the expression levels of the candidate gene biomarkers in RA patients was constructed and evaluated using a validation cohort.

**Results:**

Six candidate gene biomarkers (*MX1, OASL, SPINK1, CRK, GRAPL* and *RNF2*) were identified to be predictors of TG therapy. Following the construction of a PLS-based model using their expression levels in peripheral blood, both the 5-fold cross-validation and independent dataset validations showed the high predictive efficiency of this model, and demonstrated a distinguished improvement of the PLS-model based on six candidate gene biomarkers’ expression in combination over the commonly used clinical and inflammatory parameters, as well as the gene biomarkers alone, in predicting RA patients’ response to TG tablets.

**Conclusions:**

This hypothesis-generating study identified *MX1, OASL, SPINK1, CRK, GRAPL* and *RNF2 as* novel targets for RA therapeutic intervention, and the PLS model based on the expression levels of these candidate biomarkers may have a potential prognostic value in RA patients treated with TG tablets.

**Electronic supplementary material:**

The online version of this article (10.1186/s12967-018-1549-9) contains supplementary material, which is available to authorized users.

## Background

*Tripterygium wilfordii* Hook F (TwHF), a traditional Chinese medicine, has been used in the treatment of rheumatoid arthritis (RA) for hundreds of years in China [1]. TwHF has been considered as a potential source for developing new drugs to treat RA, due to its considerable improvement in the outlook for patients suffering from RA [2]. Tripterysium glycosides (TG) tablets, as the main effective ingredients of TwHF, are the most commonly used TwHF-based therapy and display better therapeutic effects than several first-line disease-modifying anti-rheumatic drugs according to recent clinical observations [3]. However, approximately 30% of RA patients treated with TG tablets fail to achieve clinical improvement, depicting significant inter-individual variations caused by epigenetic, physiologic, environmental and especially genetic factors in combination or alone [4, 5]. The heterogeneity of RA patients in pathological manifestations, disease progression and treatment response implies the existence of several disease subtypes at the molecular level [6]. Researchers are trying to figure out various biomarkers of therapeutic response in rheumatic diseases for further benefiting individualized treatment and curative outcomes, including microRNAs, genes, proteins and etc. [7]. However, existent biomarkers that reflect bone and cartilage turnover are insufficient surrogates for predicting therapeutic outcomes of certain drugs [8]. Thus, it is of great clinical significance to identify biomarkers and to develop tools, which are predictive of RA patients’ response to TG tablets.

**Fig. 1:**
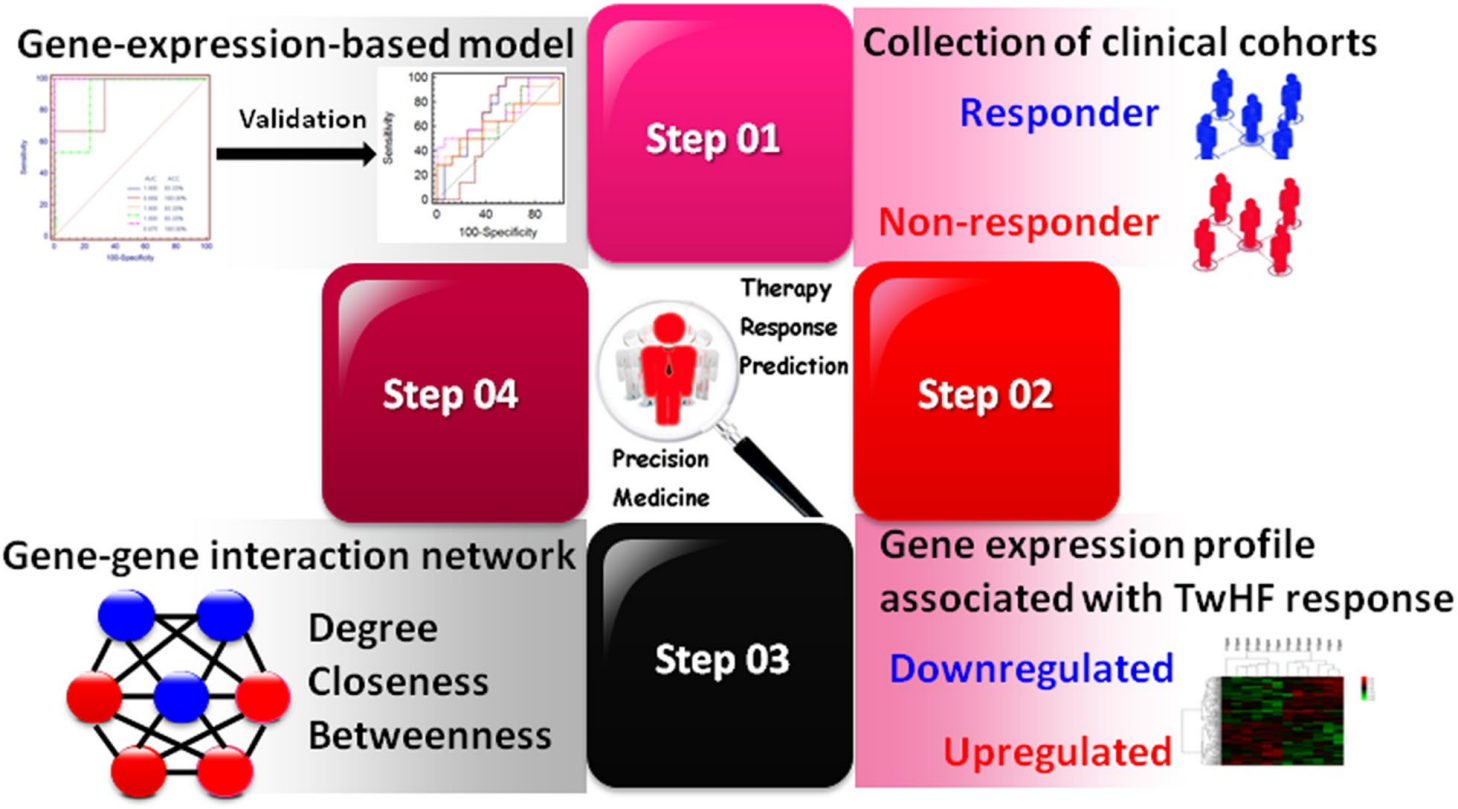
A schematic diagram of the systematic strategies to identify gene biomarkers and to construct PLS-based model that predictive of response to TG tablets

In recent years, high-capacity genomics and transcriptomics technologies, such as gene microarrays that detect expression profiles of numerous genes simultaneously and comprehensively, play important roles in identifying biomarkers for disease behavior and therapeutic response prediction [9, 10]. Although gene expression microarray is characterized with high sensitivity and high-throughput, it is insufficient to comprehensively clarify the entire biological regulatory processes in RA. Moreover, variations in sample size and quality may often result in the inconsistencies among numerous differentially expressed genes identified by microarrays [11]. To address these problems, we herein designed a hypothesis-generating study combining the high-throughput merits of gene expression profiling and the comprehensive illustration of drug-disease interactions by molecular network analysis, to assess the differentially expressed genes between responsive and non-responsive RA patients to TG tablets and to identify the candidate gene biomarkers according to both the differential expression patterns and the network topological features, as well as to construct a partial-least-squares (PLS) model based on the expression levels of the candidate gene biomarkers in peripheral blood for stratification and prediction of RA patients’ response to TG tablets (Fig. 1).

## Methods

### Ethics statement

This study was performed according to the guidelines of the Declaration of Helsinki for humans and was approved by the Research Ethics Committee of Guang’anmen Hospital. The informed consent was obtained from all patients.

### Patients

Two RA patient cohorts, a discovery cohort (n = 12, 6 responders and 6 non-responders, collected from January 2015 to December 2015 in Division of Rheumatology, Guang’anmen Hospital) and a validation cohort (n = 31, 15 responders and 16 non-responders, collected from January 2016 to June 2017 in Division of Rheumatology, Guang’anmen Hospital), were enrolled into this study apart. The discovery cohort was used to detect the gene expression profile in peripheral blood mononuclear cells (PBMCs) and to train PLS model predictive of response to TG tablets. The validation cohort was used to validate the expression levels of candidate gene biomarkers of response to TG tablets by qPCR assay and to evaluate the predictive efficiency of PLS-based model in an independent dataset test. Inclusion Criteria of the two cohorts included (1) a diagnosis of active RA based on the American College of Rheumatology (ACR) 1987 criteria for RA or the 2010 ACR/European League against Rheumatism (EULAR) Criteria [12]; (2) a symptom duration of less than 1 year; (3) no use of DMARDs previously; (4) Availability of clinical and laboratory parameters at initiation of TG tablets (purchased from Zhejiang Deengde Co., Ltd., Z33020422, Xinchang, Zhejiang) and after 12 weeks, as well as availability of peripheral blood samples. Patients received oral TG tablets (20mg.tid.po) for 12 weeks. Responders to TG tablets were defined as patients who were treated with TG tablets for 12 weeks achieved ACR 20, and non-responders were defined as patients who were treated with TG tablets for 12 weeks but not achieved ACR 20 [13]. There are no significant differences in clinical and laboratory parameters between the cohorts (Table 1 and Additional file 1).

**Table 1:** Clinical and laboratory parameters of RA patients enrolled in the current study

Parameters	Discovery cohort (n = 12)	Validation cohort (n = 31)
Age (years, mean ± SD)	55.9 ± 10.4	57.9 ± 12.0
Gender (male/female)	2/10	8/23
Disease duration (months, mean ± SD)	36.5 ± 30.6	41.5 ± 21.5
Erythrocyte sedimentation rate (ESR, mm/H, mean ± SD)	58.4 ± 21.3	40.5 ± 27.3
C-reactive protein (CRP, mg/dL, mean ± SD)	25.6 ± 22.9	21.5 ± 36.7
Positive rheumatoid factor (n, %)	10, 83.3	24, 77.4
Positive anti-cyclic citrullinated peptide (CCP) antibodies (n, %)	9, 75	24, 77.4

### Gene expression profiling

Peripheral blood samples (1.5 mL) were collected from RA patients before the treatment of TG tablets in EDTA plasma tubes. Peripheral blood mononuclear cells (PBMCs) were isolated by standard Ficoll density-gradient centrifugation, and washed twice in sterile phosphate buffered saline (PBS). Total cellular RNA was isolated from PBMC using QIAGEN RNeasy Mini Kit according to the manufacturer’s protocol (Cat.No.217004, Qiagen, Hilden, Germany). Residual DNA contamination was removed using the RNase-Free DNase Set (Cat.No.79254, Qiagen, Hilden, Germany). The concentration of total RNA was measured using a NanoDrop spectrophotometer (Nanodrop technologies, Montchanin, DE, USA). Total RNA was eluted in 15 μl of RNase-free water and stored at − 80 °C.

mRNA expression profile in PBMCs obtained from responders and non-responders of TG tablets were respectively detected using Affymetrix EG1.0 array carried out by Shanghai GMINIX Biotechnology Corporation, Shanghai, China. The mRNA expression microarray data of GSE106893 are provided in National Center of Biotechnology Information Gene Expression Omnibus (https://www.ncbi.nlm.nih.gov/geo/query/acc.cgi?acc=GSE106893).

### Differentially expressed mRNAs screening

mRNAs with significantly differential expression between responder and non-responder groups were identified using the criteria of |log2 fold change (FC)| > 0.5 and P value < 0.05 by the RVM *t*-test. The heat map package in R (version 1.0.2, R Core Team, Vienna, Austria) was used for the hierarchical clustering analysis.

### Gene signal transduction network analysis

Gene signal transduction network associated with the response to TG tablets was constructed using links among differentially expressed genes between responder and non-responder groups, according to the interaction data obtained from the public database STRING (Search Tool for Known and Predicted Protein–Protein Interactions, version 10.0, http://string-db.org/) [14]. Highly reliable gene–gene interaction data with a combined evidence score higher than the median value of all scores were selected. In the network, nodes referred to differentially expressed genes between responder and non-responder groups, and edges referred to interactions between the nodes.

To identify the candidate gene biomarkers of the response to TG tablets, we evaluated the topological importance of each nodes by calculating the following four topological features: (1) node degree: the sum of connection strengths of node i with the other genes, which measures how correlated a gene is with all other genes in a network; (2) node betweenness: the importance of a node in a network relative to other nodes; (3) node closeness: measuring how long it will take to spread information from node i to all other nodes sequentially. The larger a node degree/betweenness/closeness is, the more important the node is in the network.

### Pathway enrichment analysis

To understand functions of candidate biomarkers screened by differential expression data analysis and network calculation, a pathway enrichment analysis was performed using the Database Visualization and Integrated Discovery software (DAVID, http://david.abcc.ncifcrf.gov/home.jsp, version 6.7) based on the pathway data obtained from the Kyoto Encyclopedia of Genes and Genomes database (KEGG, http://www.genome.jp/kegg/, updated on November 18, 2016) [15, 16]. Only functional annotations having the enrichment P values corrected by both algorithms Bonferroni and Benjamini (P < 0.05) were selected for further analysis.

Construction of gene-expression-signature-based PLS-model and evaluation of model performance by 5-fold cross-validation.

The PLS algorithm was used to construct a model for predicting the response to TG tablets of RA patients based on the expression levels of candidate gene biomarkers in peripheral blood. As our previous description [17], the objective criterion for constructing components in PLS is to sequentially maximize the covariance between the response variable and a linear combination of the predictors. Let ${x}$ be $n{ \times }p$ matrix of $n$ cases and $p$ candidate gene biomarkers. Also, let $y$ denote the $n{ \times }1$ vector of response values, such as the indicator of responders or non-responders. The components are constructed to maximize the objective criterion based on the sample covariance between $y$ and $x_{c}$. Thus, we find the weight vector $w$ satisfying the following objective criterion.

1 $$ {w} = {argmaxcov}^{{2}} {(x}_{{w}} {,}\,{y)} $$

Next, a training dataset was used to calculate weight coefficients of different gene biomarkers in PLS model. Six candidate gene biomarkers in PLS model are denoted as:

2 $$ {p}\,{ = }\,{\{ p}_{{i}} {\} ,}\quad {i}\,{ = }\,{1,}\,{2,}\,{3,}\,{4,}\,{5,}\,{6} $$

The score of the PLS model for each sample is defined as:

3 $$ Score\, = \,\sum {L_{{P_{i} }} \,{ \times }\,W_{{P_{i} }} } ,\quad i\, = \,1,\,2,\,3,\,4,\,5,\,6 $$

where ${L}_{{{P}_{{i}} }}$ refers to the expression level of the candidate gene biomarker ${p}_{{i}}$ in each RA patient.

Then, the training dataset was used to input the PLS model so as to calculate the threshold value $T$ of score by selecting the cutoff value on which the area under receiver operating characteristic (ROC) curve ($AUC$) was the biggest. Finally, the PLS classifier decides: if $Score\,\text{ > }\,T$, the sample can be predicted as the responder of TG tablets.

For 5-fold cross-validation, the expression levels of six candidate gene biomarkers in the discovery cohort was divided into two parts: training dataset and testing dataset. Due to the small sample size of the discovery cohort, the 5-fold cross-validation (five times) was performed. The average $Accuracy$,${Sensitivity}$ and ${Specificity}$, as well as the average area-under-curve ($AUC$) from receiver-operating-characteristic (ROC) curves were calculated as the following formula:

4 $$ {Sensitivity}\,{ = }\,\frac{{{TP}}}{{{TP}\,{ + }\,{FN}}} $$

5 $$ {Specificity}\,{ = }\,\frac{{{TN}}}{{{TN}\,{ + }\,{FP}}} $$

6 $$ {Accuracy}\,{ = }\,\frac{{\sum {{TP}} \, + \,{TN}}}{{N}} $$

where $TP$, $TN$, $FP$, $FN$ respectively refer to the number of true positive, true negative, false positive and false negative result components in a test, while $N$ refers to the total number of predicted samples.

### Quantitative PCR analysis

To evaluate the predictive performance of our gene-expression-signature-based PLS-model using independent dataset test, quantitative PCR analysis for six candidate genes in the model was performed using the peripheral blood samples obtained from the validation cohort according to our previous studies [18, 19]. GAPDH and RPS18 were used as two internal controls for candidate gene expression normalization and quantification. Quantitative PCR analysis and data collection were performed on the ABI 7900HT qPCR system using the primer pairs listed in Additional file 2. Relative quantification of gene expression was evaluated using the comparative cycle threshold (CT) method. The raw quantifications were respectively normalized to GAPDH and RPS18 values for each sample and fold changes were shown as mean ± SD in three independent experiments with each triplicate.

### Evaluation of model performance by independent dataset test

The differential expression patterns of candidate gene biomarkers and the performance of our gene-expression-signature-based PLS-model that were predictive of response to TG tablets were both validated by the independent dataset test using the validation cohort. The accuracy and area under ROC curves (AUC) were calculated as formula 4–6.

### Statistical analyses

Statistical analyses were performed using SPSS software (Version 13.0, Statistical Program for Social Sciences, Inc: Chicago, IL, USA). Expression levels of candidate gene biomarkers between the responder and non-responder groups were compared by one-way analysis of variance. *P*-values < 0.05 were considered significant.

## Results

### Differentially expressed genes associated with response to TG tablets

The differentially expressed genes associated with the response to TG tablets were evaluated by comparing the gene expression profiles between responder and non-responder groups. A total of 212 genes (102 upregulated and 110 downregulated) showed significantly differential expression levels between the two groups (all fold change > 1.2 and P < 0.05). The differentially expressed genes were listed in Additional file 3.

**Fig. 2:**
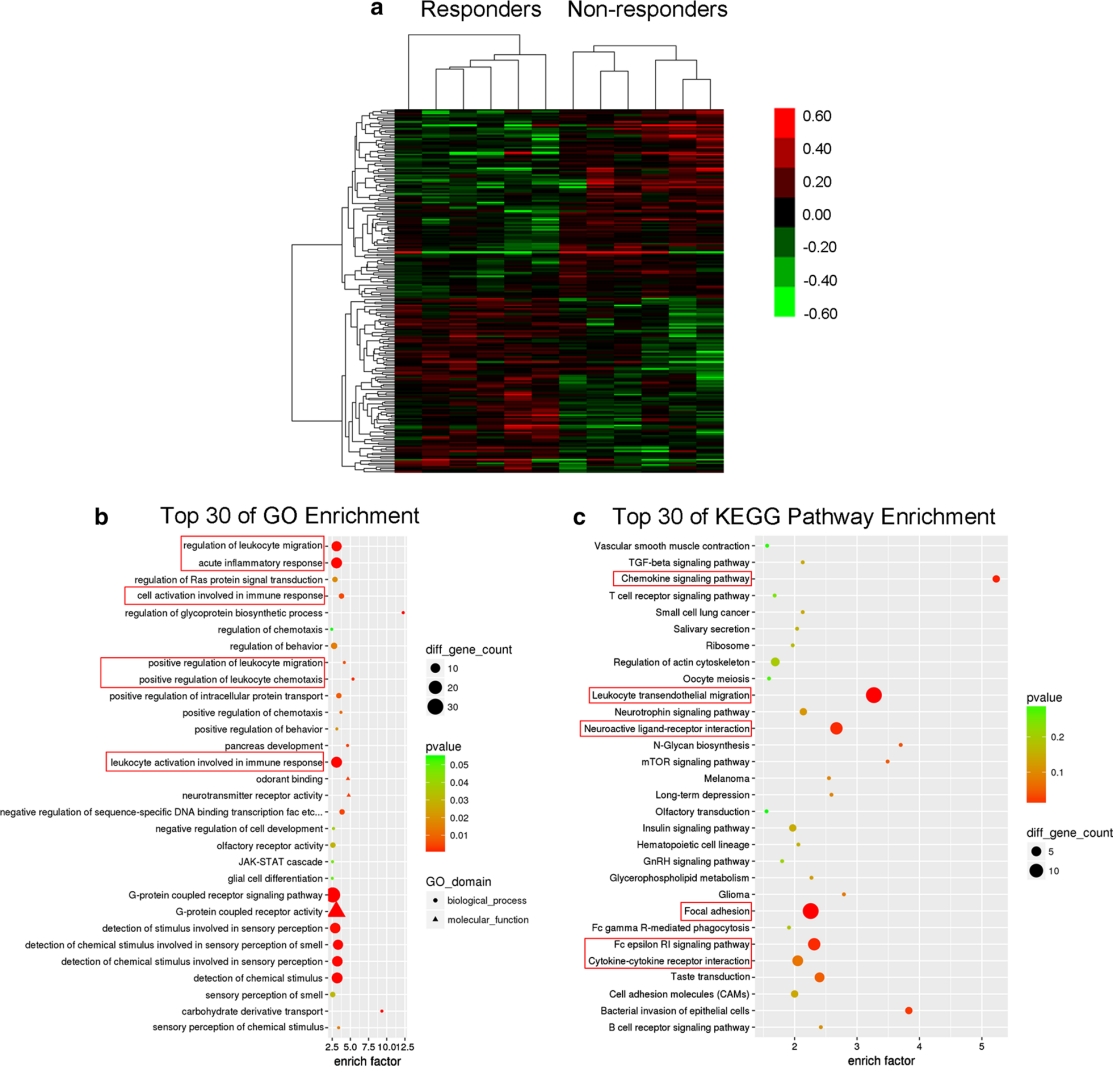
Differentially expressed genes associated with response to TG tablets. **a** Heat map showing hierarchical clustering of mRNAs, whose expression changes were more than 1.5-fold in the comparison between the responder and non-responder groups. In clustering analysis, up- and down-regulated genes are colored in red and green, respectively. **b**, **c** Top 30 of GO items and KEGG pathways enriched by the differentially expressed genes in the comparison between the responder and non-responder groups. GO items and KEGG pathways with red marks were significantly associated with RA development and progression

In addition, the heat-maps (Fig. 2a) and the unsupervised hierarchical clustering of the above differentially expressed gene profiles revealed distinctive patterns for responders and non-responders to TG tablets. According to the pathway enrichment analysis, the differentially expressed genes associated with response to TG tablets were significantly enriched in the biological processes and pathways of inflammatory response and immune response, including acute inflammatory response, leukocyte activation involved in immune response, cell activation involved in immune response, chemokine signaling pathway, leukocyte transendothelial migration, positive regulation of leukocyte migration, positive regulation of leukocyte chemotaxis, leukocyte activation involved in immune response, regulation of leukocyte migration, Fc epsilon RI signaling pathway and cytokine–cytokine receptor interaction (all P < 0.05, Fig. 2b, c). Growing evidence show that the pathogenesis of RA may be related to the defects in immune-modulation and a host of inflammatory mechanisms [20–22], thus, the differentially expressed genes, such as *CRK*, *GHR*, *RNF2*, *RNF8*, *VAV2*, which are involved into these biological processes might influence the therapeutic effects of TG tablets in controlling inflammation response and regulating immunity during RA progression. Moreover, several differentially expressed genes, such as *CRK*, *VAV2*, *IGF1*, were significantly associated with focal adhesion (Fig. 2c), which has been proved to promote the proliferation, migration and invasion of synovial cells, and contributes to the occurrence and development of RA pathological changes [23].

### Identification of candidate gene biomarkers that predict response to TG tablets based on the discovery cohort

**Fig. 3:**
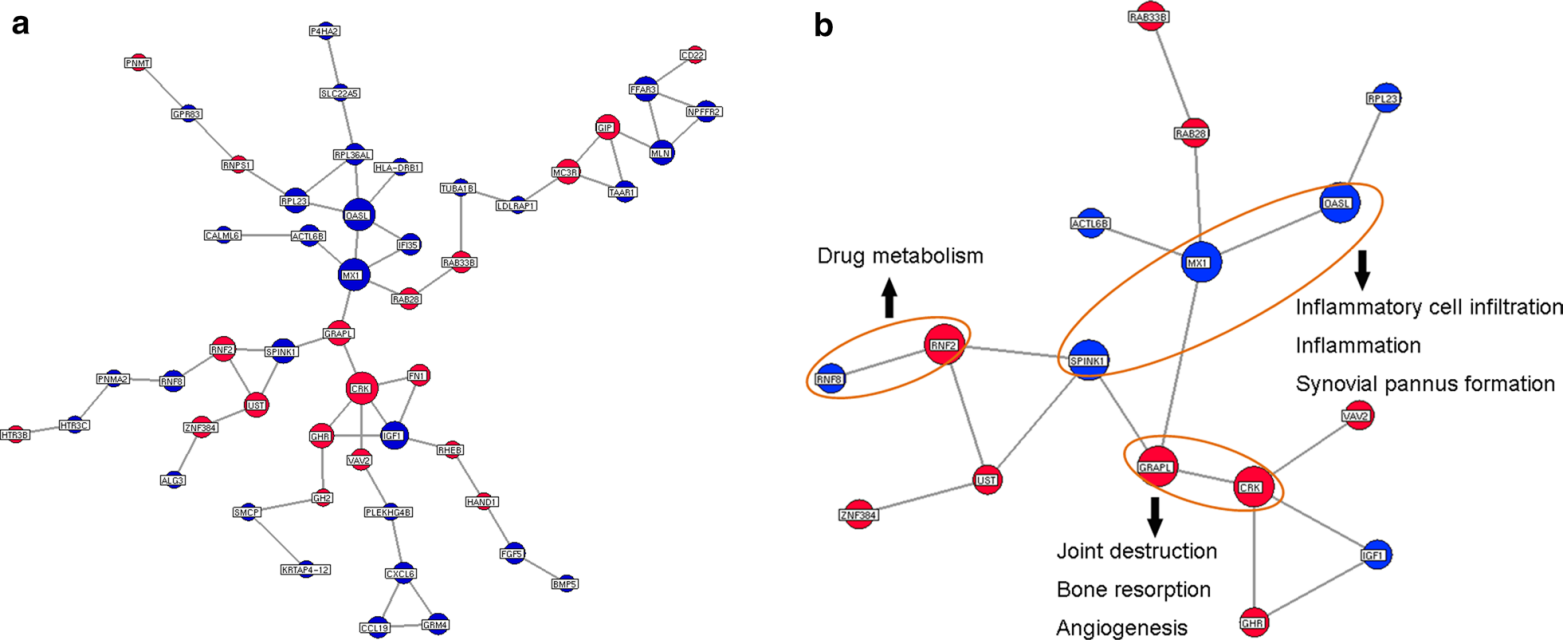
Gene signal transduction network associated with patients’ response to TG tablets. **a** Gene signal transduction network based on the interaction data of 212 differentially expressed genes obtained from STRING database. Red and blue circle nodes respectively refer to the upregulated and downregulated mRNAs in the responder group compared to the non-responder group. The node sizes represented the degree values according to the ascending order. **b** The 16 major genes’ sub-module constructed using the direct interactions among them. Red and blue circle nodes respectively refer to the upregulated and downregulated mRNAs in the responder group compared to the non-responder group. The node sizes represented the fold-change of gene expression levels in responder and non-responder groups according to the ascending order

To identify the candidate gene biomarkers that predict response to TG tablets, the gene–gene interaction data of 212 differentially expressed genes were obtained from STRING database and the gene signal transduction network was constructed as shown in Fig. 3a. Following the calculation of degree, closeness and betweenness centrality values, 16 genes with the three feature values higher than the corresponding median values simultaneously were identified as the major genes with great topological importance in the gene–gene interaction network associated with response to TG tablets (Table 2). In addition, the 16 major genes’ sub-module (Fig. 3b) was constructed using the direct interactions among them, implying that these major genes had the closed links with each other in the network. According to differential expression levels of the 16 major genes in the comparison between responder and non-responder groups, *MX1*, *OASL*, *SPINK1*, *CRK*, *GRAPL* and *RNF2* showed more significant dysregulation [$Fold - change_{(responder/non - responder)} \, > \, 1. 5$ and *P *< 0.05] than other genes (Table 2). Moreover, according to the database of GeneCards (http://www.genecards.org/, Version 4.5.1), the six candidate gene biomarkers were functionally involved into several signal pathways associated with major pathological events during RA progression, such as inflammatory cell infiltration, inflammation, synovial pannus formation, angiogenesis, joint destruction and bone resorption, as well as drug metabolism (Table 3 and Fig. 3b). Considering their significantly differential expression patterns, great network topological importance and functional relevance to RA, we selected *MX1*, *OASL*, *SPINK1*, *CRK*, *GRAPL* and *RNF2* as the candidate gene biomarkers and their expression levels in peripheral blood would be used to construct the PLS-based model for predicting the response to TG tablets.

**Table 2:** Network topological features and differential expression patterns of 16 major genes associated with RA patients’ response to TG tablets

Gene	Network topological features			Differential expression patterns		
	Degree	Closeness	Betweenness	P_value	Fold_change	Style
ACTL6B	2	2.69	1.37	0.04	0.82	Down
*CRK*	*5*	*2.71*	*7.99*	*0.02*	*1.52*	*Up*
GHR	3	2.69	1.37	0.03	1.25	Up
*GRAPL*	*3*	*2.73*	*12.48*	*0.02*	*1.59*	*Up*
IGF1	4	2.69	1.43	0.02	0.81	Down
*MX1*	*5*	*2.72*	*11.21*	*0.02*	*0.55*	*Down*
*OASL*	*5*	*2.70*	*5.02*	*0.01*	*0.62*	*Down*
RAB28	2	2.69	2.64	0.02	1.22	Up
RAB33B	2	2.66	1.37	0.02	1.24	Up
*RNF2*	*3*	*2.68*	*2.64*	*0.02*	*1.65*	*Up*
RNF8	2	2.66	1.37	0.04	0.80	Down
RPL23	3	2.67	1.37	0.04	0.79	Down
*SPINK1*	*3*	*2.71*	*6.66*	*0.02*	*0.63*	*Down*
UST	3	2.68	2.64	0.04	1.23	Up
VAV2	2	2.68	1.37	0.03	1.21	Up
ZNF384	2	2.66	1.37	0.01	1.23	Up

The italic marks refer to the candidate gene biomarkers with high topological importance and high fold-change (> 1.5) of expression levels in responder and non-responder groups

**Table 3:** Candidate gene biomarkers and the involved RA-related pathways

Gene biomarkers	Pathways	Relevance to RA
MX1	Cytokine signaling in immune system	Inflammatory cell infiltration Inflammation Synovial pannus formation
	Innate immune system	
	Peginterferon alpha-2a/peginterferon alpha-2b pathway (Hepatocyte), pharmacodynamics	Drug metabolism
OASL	Cytokine signaling in Immune system	Inflammatory cell infiltration Inflammation Synovial pannus formation
	Innate immune system	
	Immune response IFN alpha/beta signaling pathway	
	Interferon signaling	
RNF2	Cellular senescence	Drug metabolism
	SUMOylation of DNA damage response and repair proteins	
	Metabolism of proteins	
	Chromatin regulation/acetylation	
	DNA damage	
SPINK1	Regulation of peptidase activity	Inflammatory cell infiltration Inflammation Synovial pannus formation
	Nitric oxide mediated signal transduction	
	Regulation of peptidyl-tyrosine phosphorylation	
CRK	RET signaling	Angiogenesis
	MET promotes cell motility	Joint destruction
	Focal adhesion	Bone resorption
	Integrin alphaIIb beta3 signaling	Angiogenesis
GRAPL	RET signaling	Angiogenesis

### Validation of candidate gene biomarkers that predict response to TG tablets based on the validation cohort

**Fig. 4:**
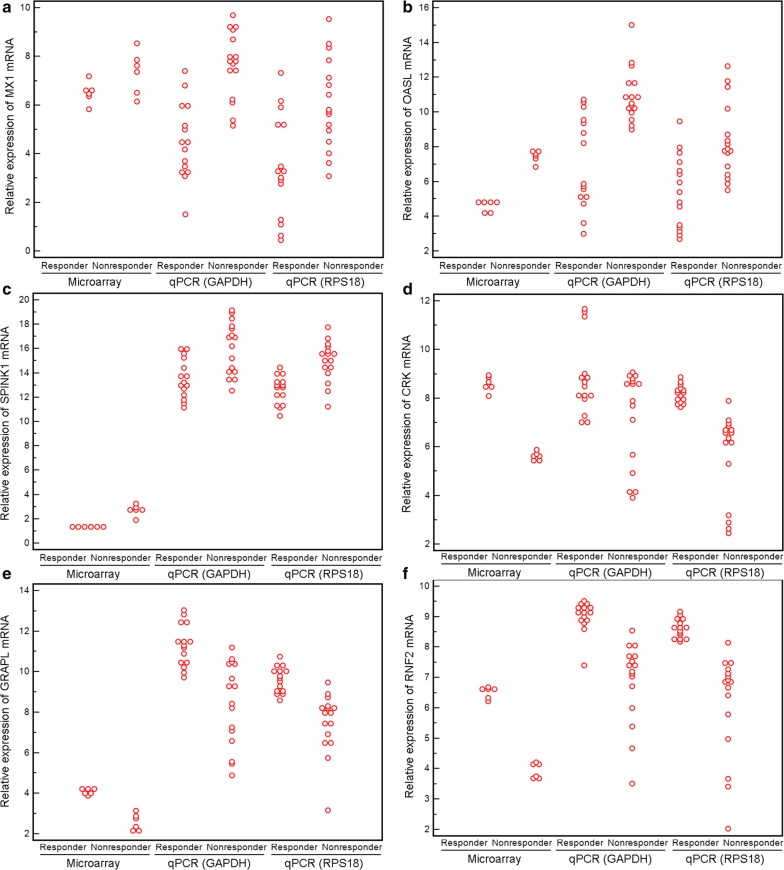
Expression levels of the six candidate gene biomarkers (**a** for MX1; **b** for OASL; **c** for SPINK1; **d** for CRK; **e** for GRAPL; **f** for RNF2) detected by microarray and quantitative PCR analyses. Each dot displayed the expression levels of the six candidate gene biomarkers in each individual patients (n = 6 for each group in the discovery cohort; n = 16 and 15 for non-responder and responder groups in the validation cohort, respectively)

Following the identification of the six most recognized genes (*MX1*, *OASL*, *SPINK1*, *CRK*, *GRAPL and RNF2*) as candidate biomarkers of response to TG tablets, we tried to validate the microarray data using the validation cohort of 31 patients by quantitative PCR analysis. Consistently, the expression levels of *MX1*, *OASL* and *SPINK1* in peripheral blood obtained from the responders were significantly lower than the non-responders based on both two internal controls *RPS18* and *GAPDH* (all P < 0.001, Fig. 4a–c). In addition, the expression levels of *CRK*, *GRAPL* and *RNF2* were markedly elevated in the responders compared with the non-responders, which were also in line with the microarray data (all P < 0.001, Fig. 4d, e).

### The PLS-based model efficiently predicts response to TG tablets

The PLS-based model using expression levels of the six gene biomarkers (*MX1*, *OASL*, *SPINK1*, *CRK*, *GRAPL* and *RNF2*) was constructed. The discovery cohort was used to determine the weight value of each gene biomarker and the threshold of the model. As a result, the weight values of *MX1*, *OASL*, *SPINK1*, *CRK*, *GRAPL* and *RNF2* were respectively − 0.4694, − 0.2494, − 0.5592, 0.3429, 0.4054 and 0.3504, and the threshold was − 0.03.

In addition, the 5-fold cross-validation confirmed that the accuracy values of the model in the five tests were respectively 83.33, 100.00, 83.33, 83.33 and 100.00%, and the AUC values were respectively 1.000, 0.889, 1.000, 1.0000, 0.875. In the independent test validation, the expression levels of *MX1, OASL, SPINK1, CRK, GRAPL* and *RNF2* in 31 RA peripheral blood samples (normalized by GAPDH and RPS18, respectively) were used to validate the performance of our model. As a result, the accuracy and AUC values of the PLS-based model based on the expression levels of the six gene biomarkers using GAPDH as an internal control were respectively 90.32% and 0.950, which was consistent with the PLS-based model based on the expression levels of the six gene biomarkers using RPS18 as an internal control (the accuracy was 87.10% and the AUC value was 0.934). Both the 5-fold cross-validation and the independent test validation indicated the great reliability and efficacy to screen responders to TG tablets from RA patients against different test datasets.

**Fig. 5:**
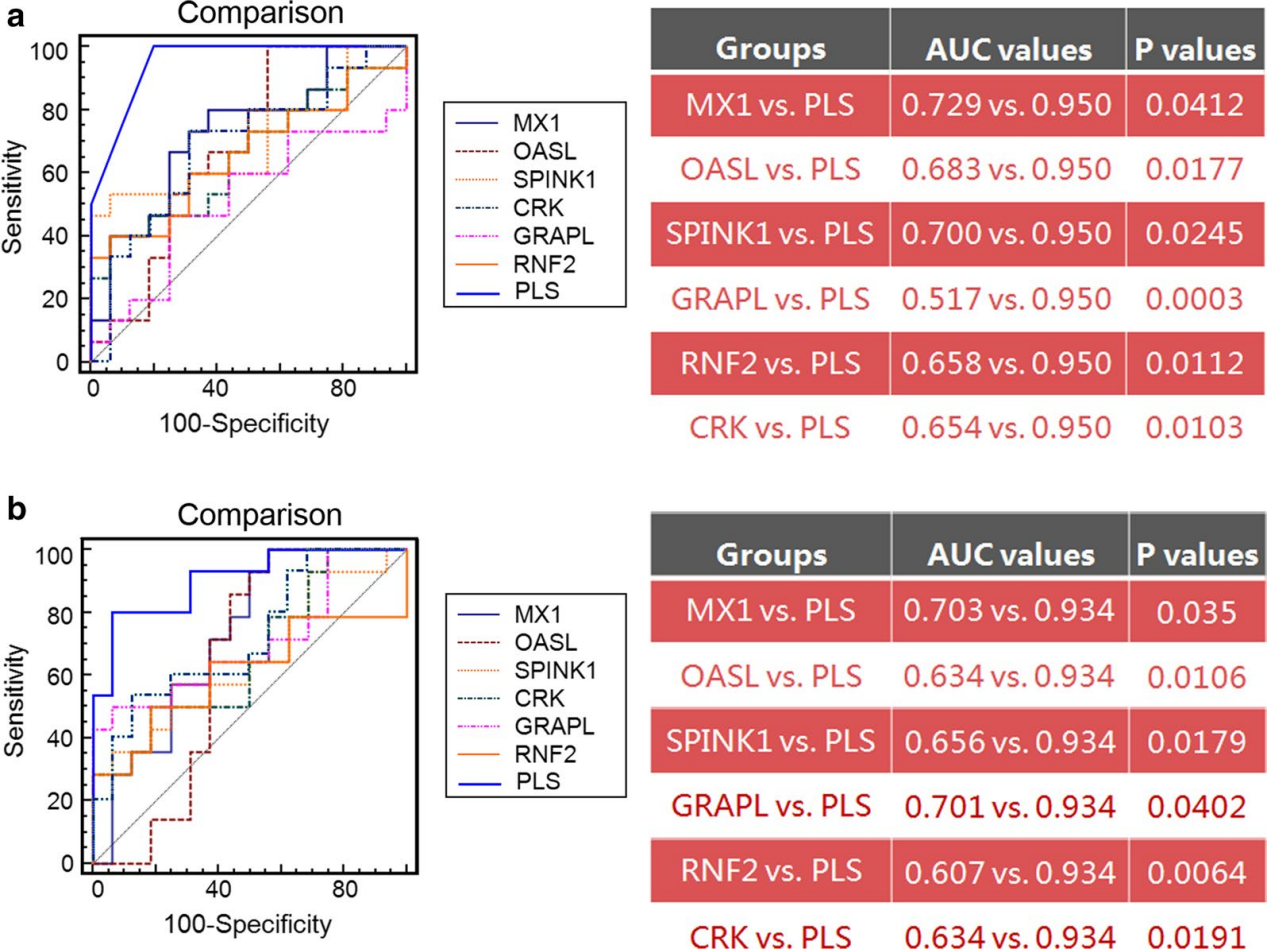
ROC comparison of the six candidate gene biomarkers alone, their average expression level with the PLS-based model in predicting response to TG tablets. **a** ROC curves and AUC comparison results of MX1, OASL, SPINK1, CRK, GRAPL, RNF2, and the average of their expression levels using GAPDH as an internal control, as well as the PLS-based model in predicting response to TG tablets. **b** ROC curves and AUC comparison results of MX1, OASL, SPINK1, CRK, GRAPL, RNF2, and the average of their expression levels using RPS18 as an internal control, as well as the PLS-based model in predicting response to TG tablets

Our PLS-based model was constructed by combining the expression levels of the six candidate gene biomarkers due to their topological importance and relevance in the gene signal transduction network associated with the response to TG tablets. To verify the rationality of this design, we firstly compared the performance of the PLS-based model with the six candidate gene biomarkers alone based on the validation cohort. As shown in Fig. 5, neither the expression level of the single gene biomarker nor their average level showed better power in predicting response to TG tablets than the PLS-based model which was constructed based on the expression levels of all six candidate gene biomarkers using GAPDH and RPS18 as internal controls.

**Fig. 6:**
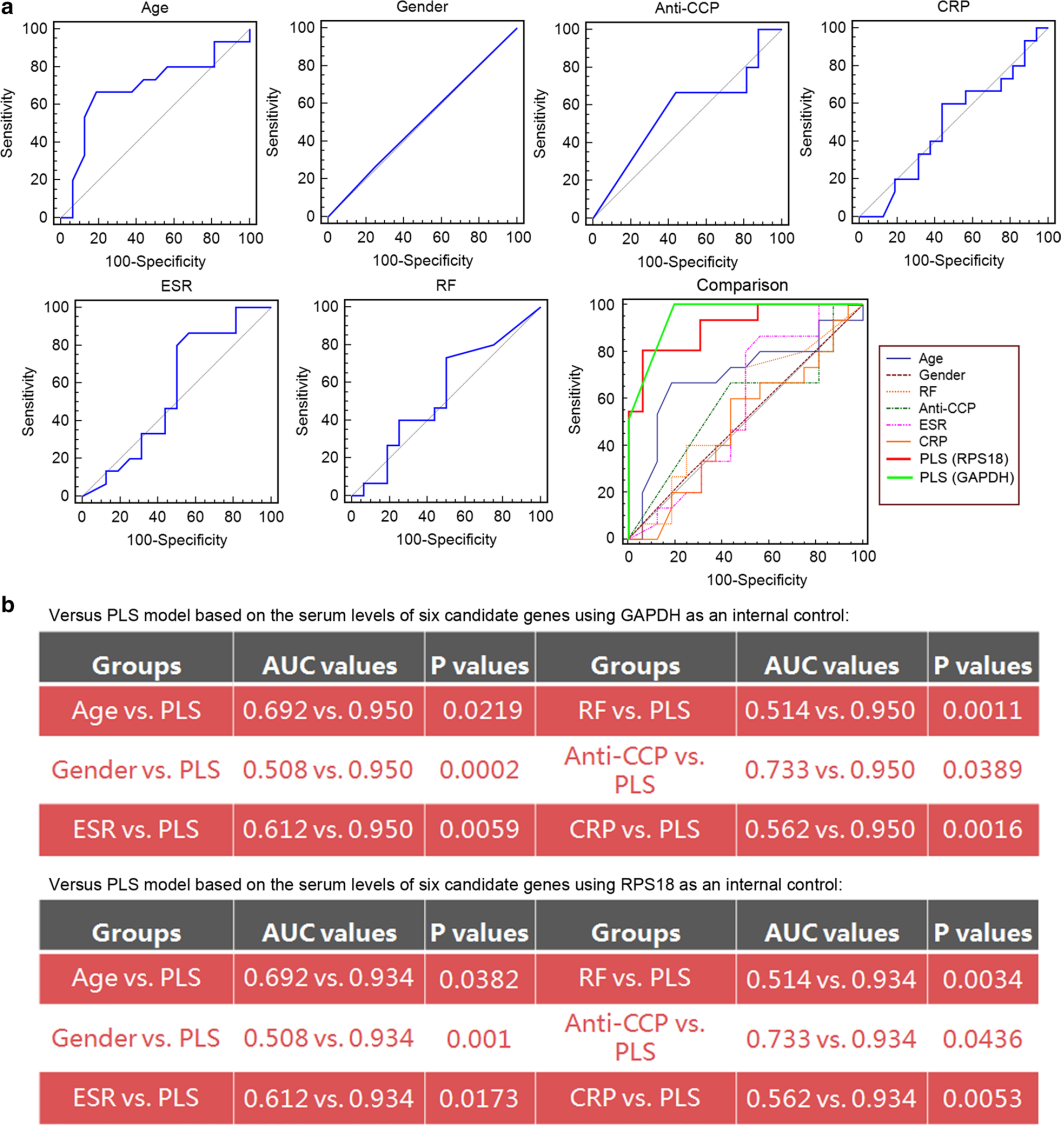
ROC comparison of the six commonly used clinical and inflammatory parameters of RA with the PLS-based model in predicting response to TG tablets. **a** ROC curves of patients’ age, gender, ESR, CRP, RF and anti-CCP, as well as their comparisons with the PLS model based on the expression levels of the six candidate genes using GAPDH and RPS18 as internal controls. **b** Statistical analysis of AUC comparison between the six commonly used clinical and inflammatory parameters of RA, and the PLS model in predicting response to TG tablets

To determine the advantage of our PLS-based model in predicting response to TG tablets over various commonly used clinical and inflammatory parameters of RA, we compared its prediction efficacy with patients’ age, gender, erythrocyte sedimentation rate (ESR), as well as levels of C-reactive protein (CRP), rheumatoid factor (RF) and anti-cyclic citrullinated peptide (CCP) antibodies. ROC comparison analysis demonstrated the marked better performance of the PLS model based on the expression levels of the six candidate gene biomarkers than the clinical and inflammatory parameters (AUC value comparisons, all P < 0.05, Fig. 6).

## Discussion

Since the clinical observations have indicated that not all RA patients benefit to the same extent from the treatment of TG tablets, the identification of drug response biomarkers and the development of predictive tools based on the biomarkers may be of great significance to determine patients with a low probability of response to TG tablets, allowing clinicians to choose alternative drugs at an earlier stage of the disease without any delay of efficacious treatment. In the current study, we integrated the drug response-related gene expression profile and gene–gene interactions to identify the six candidate gene biomarkers that are predictive of response to TG tablets. Then, we applied the PLS algorithm to construct the prediction model for the treatment outcome based on the expression levels of these candidate gene biomarkers. Both 5-fold cross-validation and the independent dataset validations showed the high predictive accuracy (87.50–100.00%) and area under ROC curve (0.875–1.000) of this model. More importantly, our data demonstrated a distinguished improvement of the PLS-model based on the expression levels of the six candidate gene biomarkers in combination over the commonly used clinical and inflammatory parameters, as well as the gene biomarkers alone, in predicting RA patients’ response to TG tablets. To the best of our knowledge, this is the first study that identified the gene biomarkers of RA patients’ response to TG tablets and also evaluated their utility in the prediction model of the clinical treatment outcome.

To facilitate the implementation of personalized medicine and the improvement of therapeutic outcomes of patients with RA, recent studies have been trying to identify various predictors of response to therapy, including single nucleotide polymorphisms, genes, proteins and microRNAs, based on genomics, transcriptomics, proteomics and miRNomics, respectively [2, 24–26]. However, the studies only based on the “omics” data analysis may fail to fully explain all of the alterations occurring during the RA progression. The need for a deeper understanding of how molecular regulatory networks function in response to therapy may lead to increased efforts to model multiple “omics” dimensions simultaneously. In the context, we here performed a systematic integration of the differential expression analysis on microarray data and topological features of gene signal transduction network, which offers us two main advantages: first, it enables us to sufficiently utilize the gene co-expression information provided by the microarray data, which is believed to be more informative than expression changes of individual genes for target gene identification. Second, network analysis is a powerful tool to understand disease occurrence and progression, as well as therapies and drug responses. By integrating the topological features of biological network, some information lost in the differential expression analysis may be added to the identification of the candidate gene biomarkers predictive of response to TG tablets. Functionally, as shown in Table 3, these candidate gene biomarkers were associated with RA pathogenesis and drug metabolism. Especially, MX1 and OASL both function as interferon (IFN-I)-inducible genes and are predominantly involved into signal pathways in the immune system. Recent studies have indicated that the IFN-I signature in RA may display clinical relevance in relation to disease onset and therapeutic response [27, 28]. Sanayama et al. [29] identified MX2 (a member in the same family with MX1), and OASL as biomarkers for predicting the therapeutic response to tocilizumab in patients with RA. RNF2 (the RING domain-containing E3 ubiquitin-protein ligases RING finger protein 2) is involved in the maintenance of histone H2A levels and impacts transcriptional activity [30]. RING E3 ligases have been revealed to be involved into the control of multiple cellular processes and also regarded as candidate therapeutic target of many human diseases, including RA [31]. The ubiquitin/proteasome protein degradation pathways were found to offer a contribution to prolonging the survival of synovial fibroblasts in RA tissue [32]. Interestingly, Torre et al. [33] found that a E3 ubiquitin ligase might positively regulate type I interferon responses and promote pathogenesis during neuroinflammation, implying several possible associations of RNF2 with MX1 and OASL. Crk family adaptors have been reported to be widely expressed and mediate the timely formation of signal transduction protein complexes upon a variety of extracellular stimuli. Hisakawa et al. [34] found that tyrosine phosphorylation of Crk-associated substrate lymphocyte-type (Cas-L) was markedly enhanced in synovial fluid T cells from patients with RA. Miyake-Nishijima et al. [35] demonstrated the overexpression of Cas-L protein and the increase of its tyrosine phosphorylation in RA mouse model, as well as observed a large number of Cas-L-positive lymphocytes infiltrating to the inflammatory lesions of RA patients, implying the important roles of Cas-L in the pathophysiology of RA. The above literature reports support the evidence that the candidate gene biomarkers identified in the current study may be associated with disease progression and treatment outcome of RA.

To determine the clinical utility of the candidate gene biomarkers, we established a PLS-based prediction model for the treatment of TG tablets using the expression levels of the six candidate gene biomarkers in patients with RA. PLS is a method for modeling a relationship between two sets of multivariate data via a latent space, and of performing least squares regression in that space. It maps input and output variables to low-dimensional spaces so that the covariance of data in the latent spaces is maximized. The algorithm can efficiently differentiate the two datasets by extracting effective information from a large number of features [36–38]. Notably, both the cross-validation and the independent clinical cohort validation suggested that this model may be capable to predict the therapeutic effectiveness in RA patients treated with TG tablets, and also confirmed the essentiality of the model construction by comparing the performance of commonly used clinical and inflammatory factors, each gene biomarker alone and the model with gene combination.

## Conclusions

This hypothesis-generating study identified MX1, OASL, SPINK1, CRK, GRAPL and RNF2 as novel targets for RA therapeutic intervention, and the PLS model based on their expression levels may have the potential prognostic value in RA patients who are treated with TG tablets. Our PLS model may not only warrant evaluation in prospective studies, but also potentially benefits individualized therapy of RA in a daily clinical setting. However, the clinical cohort using in this study is the relatively small for the generation and validation of the predictive model, which may lead to some model over-fitting. Therefore, this is a hypothesis-generating study and future studies based on large clinical cohorts to verify the utility of the six gene biomarkers and the PLS model in predicting and monitoring TwHF-based treatment outcome are required.

## Additional files

**Additional file 1.** Detailed information of clinical and inflammatory parameters of RA patients enrolled in the current study.

**Additional file 2.** Primer sequences used in the qPCR analysis.

**Additional file 3.** Differentially expressed genes between responder and non-responder groups.

## Abbreviations

TwHF: *Tripterygium wilfordii* Hook F
RA: rheumatoid arthritis
DMARD: disease-modifying anti-rheumatic drug
PBMCs: peripheral blood mononuclear cells
TG: tripterysium glycosides tablets
PLS: partial-least-squares

## References

[1] Tao X, Lipsky PE (2000). The Chinese anti-inflammatory and immunosuppressive herbal remedy *Tripterygium wilfordii* Hook F. Rheum Dis Clin North Am..

[2] Jiang M, Zha Q, Zhang C, Lu C, Yan X, Zhu W, Liu W, Tu S, Hou L, Wang C, Zhang W, Liang Q, Fan B, Yu J, Zhang W, Liu X, Yang J, He X, Li L, Niu X, Liu Y, Guo H, He B, Zhang G, Bian Z, Lu A (2015). Predicting and verifying outcome of *Tripterygium wilfordii* Hook F. based therapy in rheumatoid arthritis: from open to double-blinded randomized trial. Sci Rep..

[3] Ernst E, Posadzki P (2011). Complementary and alternative medicine for rheumatoid arthritis and osteoarthritis: an overview of systematic reviews. Curr Pain Headache Rep.

[4] Lv QW, Zhang W, Shi Q, Zheng WJ, Li X, Chen H, Wu QJ, Jiang WL, Li HB, Gong L, Wei W, Liu H, Liu AJ, Jin HT, Wang JX, Liu XM, Li ZB, Liu B, Shen M, Wang Q, Wu XN, Liang D, Yin YF, Fei YY, Su JM, Zhao LD, Jiang Y, Li J, Tang FL, Zhang FC, Lipsky PE, Zhang X (2015). Comparison of *Tripterygium wilfordii* Hook F. with methotrexate in the treatment of active rheumatoid arthritis (TRIFRA): a randomised, controlled clinical trial. Ann Rheum Dis.

[5] Wang J, Cui M, Jiao H, Tong Y, Xu J, Zhao Y, Han M, Liu J (2013). Content analysis of systematic reviews on effectiveness of traditional Chinese medicine. J Tradit Chin Med.

[6] McInnes IB, Schett G (2017). Pathogenetic insights from the treatment of rheumatoid arthritis. Lancet.

[7] Burmester GR, Pope JE (2017). Novel treatment strategies in rheumatoid arthritis. Lancet.

[8] Cooper C, Bardin T, Brandi ML, Cacoub P, Caminis J, Civitelli R, Cutolo M, Dere W, Devogelaer JP, Diez-Perez A, Einhorn TA, Emonts P, Ethgen O, Kanis JA, Kaufman JM, Kvien TK, Lems WF, McCloskey E, Miossec P, Reiter S, Ringe J, Rizzoli R, Saag K, Reginster JY (2016). Balancing benefits and risks of glucocorticoids in rheumatic diseases and other inflammatory joint disorders: new insights from emerging data. An expert consensus paper from the European Society for Clinical and Economic Aspects of Osteoporosis and Osteoarthritis (ESCEO). Aging Clin Exp Res..

[9] Márquez A, Martín J, Carmona FD (2016). Emerging aspects of molecular biomarkers for diagnosis, prognosis and treatment response in rheumatoid arthritis. Expert Rev Mol Diagn..

[10] Smith SL, Plant D, Eyre S, Barton A (2013). The potential use of expression profiling: implications for predicting treatment response in rheumatoid arthritis. Ann Rheum Dis.

[11] Schneckener S, Arden NS, Schuppert A (2011). Quantifying stability in gene list ranking across microarray derived clinical biomarkers. BMC Med Genomics.

[12] Jansen R, Greenbaum D, Gerstein M (2002). Relating whole-genome expression data with protein-protein interactions. Genome Res.

[13] Li C, Li H (2008). Network-constrained regularization and variable selection for analysis of genomic data. Bioinformatics.

[14] Szklarczyk D, Morris JH, Cook H, Kuhn M, Wyder S, Simonovic M, Santos A, Doncheva NT, Roth A, Bork P, Jensen LJ, von Mering C (2017). The STRING database in 2017: quality-controlled protein-protein association networks, made broadly accessible. Nucleic Acids Res.

[15] Dennis G, Sherman BT, Hosack DA, Yang J, Gao W, Lane HC, Lempicki RA (2003). DAVID: database for annotation, visualization, and integrated discovery. Genome Biol.

[16] Kanehisa M, Goto S (2000). KEGG: Kyoto encyclopedia of genes and genomes. Nucleic Acids Res.

[17] Zhang Y, Wang S, Li D, Zhnag J, Gu D, Zhu Y, He F (2011). A systems biology-based classifier for hepatocellular carcinoma diagnosis. PLoS ONE.

[18] Zhang Y, Guo X, Xiong L, Yu L, Li Z, Guo Q, Li Z, Li B, Lin N (2014). Comprehensive analysis of microRNA-regulated protein interaction network reveals the tumor suppressive role of microRNA-149 in human hepatocellular carcinoma via targeting AKT-mTOR pathway. Mol Cancer.

[19] Lin ZY, Huang YQ, Zhang YQ, Han ZD, He HC, Ling XH, Fu X, Dai QS, Cai C, Chen JH (2014). MicroRNA-224 inhibits progression of human prostate cancer by downregulating TRIB1. Int J Cancer.

[20] Choy E (2012). Understanding the dynamics: pathways involved in the pathogenesis of rheumatoid arthritis. Rheumatology (Oxford).

[21] Rabquer BJ, Pakozdi A, Michel JE, Gujar BS, Haines GK, Imhof BA, Koch AE (2008). Junctional adhesion molecule C mediates leukocyte adhesion to rheumatoid arthritis synovium. Arthritis Rheumatol.

[22] Szekanecz Z, Koch AE (2016). Successes and failures of chemokine-pathway targeting in rheumatoid arthritis. Nat Rev Rheumatol.

[23] Shelef MA, Bennin DA, Yasmin N, Warner TF, Ludwig T, Beggs HE, Huttenlocher A (2014). Focal adhesion kinase is required for synovial fibroblast invasion, but not murine inflammatory arthritis. Arthritis Res Ther..

[24] Castro-Villegas C, Pérez-Sánchez C, Escudero A, Filipescu I, Verdu M, Ruiz-Limón P, Aguirre MA, Jiménez-Gomez Y, Font P, Rodriguez-Ariza A, Peinado JR, Collantes-Estévez E, González-Conejero R, Martinez C, Barbarroja N, López-Pedrera C (2015). Circulating miRNAs as potential biomarkers of therapy effectiveness in rheumatoid arthritis patients treated with anti-TNFα. Arthritis Res Ther..

[25] Fransen J, Kooloos WM, Wessels JA, Huizinga TW, Guchelaar HJ, van Riel PL, Barrera P (2012). Clinical pharmacogenetic model to predict response of MTX monotherapy in patients with established rheumatoid arthritis after DMARD failure. Pharmacogenomics..

[26] Krintel SB, Dehlendorff C, Hetland ML, Hørslev-Petersen K, Andersen KK, Junker P, Pødenphant J, Ellingsen T, Ahlquist P, Lindegaard HM, Linauskas A (2016). Prediction of treatment response to adalimumab: a double-blind placebo-controlled study of circulating microRNA in patients with early rheumatoid arthritis. Pharmacogenomics J.

[27] de Jong TD, Lübbers J, Turk S, Vosslamber S, Mantel E, Bontkes HJ, van der Laken CJ, Bijlsma JW, van Schaardenburg D, Verweij CL (2016). The type I interferon signature in leukocyte subsets from peripheral blood of patients with early arthritis: a major contribution by granulocytes. Arthritis Res Ther..

[28] Hua J, Kirou K, Lee C, Crow MK (2006). Functional assay of type I interferon in systemic lupus erythematosus plasma and association with anti-RNA binding protein autoantibodies. Arthritis Rheumatol.

[29] Sanayama Y, Ikeda K, Saito Y, Kagami S, Yamagata M, Furuta S, Kashiwakuma D, Iwamoto I, Umibe T, Nawata Y, Matsumura R, Sugiyama T, Sueishi M, Hiraguri M, Nonaka K, Ohara O, Nakajima H (2014). Prediction of therapeutic responses to tocilizumab in patients with rheumatoid arthritis: biomarkers identified by analysis of gene expression in peripheral blood mononuclear cells using genome-wide DNA microarray. Arthritis Rheumatol..

[30] Wang H, Wang L, Erdjument-Bromage H, Vidal M, Tempst P (2004). Role of histone H2A ubiquitination in polycomb silencing. Nature.

[31] Yagishita N, Aratani S, Leach C, Amano T, Yamano Y, Nakatani K, Nishioka K, Nakajima T (2012). RING-finger type E3 ubiquitin ligase inhibitors as novel candidates for the treatment of rheumatoid arthritis. Int J Mol Med.

[32] Li F, Li X, Kou L, Li Y, Meng F, Ma F (2014). SUMO-conjugating enzyme UBC9 promotes proliferation and migration of fibroblast-like synoviocytes in rheumatoid arthritis. Inflammation..

[33] Torre S, Polyak MJ, Langlais D, Fodil N, Kennedy JM, Radovanovic I, Berghout J, Leiva-Torres GA, Krawczyk CM, Ilangumaran S, Mossman K, Liang C, Knobeloch KP, Healy LM, Antel J, Arbour N, Prat A, Majewski J, Lathrop M, Vidal SM, Gros P (2017). USP1 regulates type I interferon response and is required for pathogenesis of neuroinflammation. Nat Immunol.

[34] Hisakawa N, Tanaka H, Hosono O, Nishijima R, Ohashi Y, Saito S, Nishiya K, Hashimoto K, Morimoto C (2002). Aberrant responsiveness to RANTES in synovial fluid T cells from patients with rheumatoid arthritis. J Rheumatol.

[35] Miyake-Nishijima R, Iwata S, Saijo S, Kobayashi H, Kobayashi S, Souta-Kuribara A, Hosono O, Kawasaki H, Tanaka H, Ikeda E, Okada Y, Iwakura Y, Morimoto C (2003). Role of Crk-associated substrate lymphocyte type in the pathophysiology of rheumatoid arthritis in tax transgenic mice and in humans. Arthritis Rheumatol..

[36] Yoshida K, Shimizu Y, Yoshimoto J, Takamura M, Okada G, Okamoto Y, Yamawaki S, Doya K (2017). Prediction of clinical depression scores and detection of changes in whole-brain using resting-state functional MRI data with partial least squares regression. PLoS ONE.

[37] McIntosh A, Bookstein F, Haxby JV, Grady C (1996). Spatial pattern analysis of functional brain images using partial least squares. NeuroImage..

[38] McIntosh AR, Lobaugh NJ (2004). Partial least squares analysis of neuroimaging data: applications and advances. NeuroImage..

