# Supplementary material for: A novel gene-expression-signature-based model for prediction of response to Tripterysium glycosides tablet for rheumatoid arthritis patients
Source: J Transl Med. 2018 Jul 4;16:187. doi: 10.1186/s12967-018-1549-9 (PMC6032531; doi:10.1186/s12967-018-1549-9)
Supplement: Supplementary file 1 — Additional file 1. Detailed information of clinical and inflammatory parameters of RA patients enrolled in the current study. [file 12967_2018_1549_MOESM1_ESM.docx]

**Additional file 1 Detailed information of clinical and inflammatory parameters of RA patients enrolled in the current study**

| **Case No.** | **Groups** | **Age (Years)** | **Gender** | **RF (U/mL)** | **Anti-CCP (IU/mL)** | **ESR (mm)** | **CRP (mg/dL)** | **Disease duration (Months)** |
| --- | --- | --- | --- | --- | --- | --- | --- | --- |
| **Discovery cohort** | | | | | | | | |
| 1 | Non-responder | 45 | Male | ＜20 | ＜25 | 81 | 83.72 | 12 |
| 2 | Non-responder | 51 | Female | 115 | 50.39 | 70 | 27.81 | 30 |
| 3 | Responder | 52 | Female | 39.9 | -- | 19 | 1.85 | 6 |
| 4 | Responder | 51 | Male | -- | -- | -- | -- | 36 |
| 5 | Non-responder | 55 | Female | ＜20 | 2829 | 57 | 32.36 | 120 |
| 6 | Responder | 80 | Female | 35.9 | ＜20 | 81 | 24.44 | 12 |
| 7 | Non-responder | 72 | Female | 60.2 | 2344.6 | 82 | 19.33 | 18 |
| 8 | Non-responder | 63 | Female | 134 | 886.08 | 58 | 44.41 | 36 |
| 9 | Responder | 52 | Female | 1040 | 134.59 | 34 | 9.25 | 36 |
| 10 | Responder | 52 | Female | 63.8 | 926.67 | 71 | 4.46 | 24 |
| 11 | Non-responder | 50 | Female | 366 | 128.43 | 53 | 17.98 | 60 |
| 12 | Responder | 48 | Female | 1460 | -- | 36 | 16.36 | 48 |
| **Validation cohort** | | | | | | | | |
| 1 | Responder | 58 | Male | 417 | 823.74 | 9 | 3.65 | 36 |
| 2 | Non-responder | 58 | Male | 68.1 | ＜25 | 60 | 76.65 | 24 |
| 3 | Non-responder | 51 | Male | 278 | 3111.03 | 13 | 10.06 | 60 |
| 4 | Non-responder | 62 | Female | ＜20 | ＜25 | 22 | 3 | 120 |
| 5 | Non-responder | 66 | Female | -- | 58.42 | -- | -- | 74 |
| 6 | Non-responder | 61 | Female | 163 | 1435.32 | 57 | 20.90 | 72 |
| 7 | Responder | 65 | Female | 45.6 | -- | 13 | 4.52 | 12 |
| 8 | Non-responder | 60 | Female | 105 | 2321.58 | 25 | 5 | 48 |
| 9 | Responder | 65 | Male | 262 | -- | 38 | 18.56 | 18 |
| 10 | Non-responder | 55 | Female | 1120 | -- | 86 | 12.51 | 12 |
| 11 | Non-responder | 53 | Female | 117 | -- | 14 | 4.3 | 24 |
| 12 | Responder | 76 | Female | 325 | -- | 44 | 6.39 | 24 |
| 13 | Responder | 65 | Female | 20.9 | 1978.21 | 17 | 1 | 50 |
| 14 | Responder | 49 | Female | 70.3 | -- | 28 | 12.47 | 18 |
| 15 | Responder | 69 | Female | 29.4 | 470.72 | 31 | 1.43 | 12 |
| 16 | Responder | 74 | Female | -- | -- | 79 | 44.65 | 32 |
| 17 | Non-responder | 50 | Female | 831 | 37.82 | -- | 20 | 60 |
| 18 | Non-responder | 56 | Female | -- | -- | 87 | 18 | 48 |
| 19 | Responder | 49 | Female | -- | -- | -- | 1 | 60 |
| 20 | Responder | 66 | Female | 447 | 2148.21 | 23 | 3.35 | 48 |
| 21 | Non-responder | 84 | Male | ＜20 | -- | 92 | 177.62 | 72 |
| 22 | Responder | 66 | Female | 25.6 | 2176.7 | 81 | 22.53 | 15 |
| 23 | Responder | 62 | Male | ＜20 | -- | 29 | 5.86 | 24 |
| 24 | Non-responder | 60 | Female | ＜20 | -- | 73 | 2.83 | 12 |
| 25 | Non-responder | 48 | Female | -- | 40.07 | 71 | -- | 36 |
| 26 | Responder | 62 | Female | 1010 | -- | 17 | 6.13 | 96 |
| 27 | Non-responder | 33 | Male | -- | -- | 10 | 17 | 18 |
| 28 | Responder | 55 | Female | 270 | -- | 28 | 20.37 | 36 |
| 29 | Responder | 25 | Male | -- | -- | 23 | 91.10 | 60 |
| 30 | Non-responder | 51 | Female | ＜20 | ＜25 | 44 | 12.07 | 30 |
| 31 | Non-responder | 40 | Female | 503 | -- | 19 | 1 | 36 |

Note: "--" refers to no information for a certain patient.
