# Supplementary material for: A novel gene-expression-signature-based model for prediction of response to Tripterysium glycosides tablet for rheumatoid arthritis patients
Source: J Transl Med. 2018 Jul 4;16:187. doi: 10.1186/s12967-018-1549-9 (PMC6032531; doi:10.1186/s12967-018-1549-9)
Supplement: Supplementary file 2 — Additional file 2. Primer sequences used in the qPCR analysis. [file 12967_2018_1549_MOESM2_ESM.pdf]

Additional file 2 Primer sequences used in the qPCR analysis

Primers	Sequence
MX1_F	CTCCGACACGAGTTCCACAA
MX1_R	GGCTCTTCCAGTGCCTTGAT
OASL_F	TTCAGCGAGCTGCAGAGAAA
OASL_R	CCCTCTGCTCCACTGTCAAG
SPINK1_F	TGACCCTGTCTGTGGGACTG
SPINK1_R	TCAGCAAGGCCCAGATTTTTG
CRK_F	AGGCAGGGTAGTGGAGTGAT
CRK_R	CTTCAGGCTTGTCCCGGATT
GRAPL_F	CTACAGAGAGCGACGAGCTG
GRAPL_R	GAAGGCTCCCAGATGGTTCC
RNF2_F	CGAACACCTCAGGAGGCAAT
RNF2_R	TGCCACTTCTAAGGGCTGTG
VAV2_F	ACGGGGAACTGAAAGTCCG
VAV2_R	CCACATTTTCCCGTGAGACTTC
GAPDH	Cat No. B661104-0001 [Sangon Biotech (Shanghai) Co., Ltd.]
RPS18	Cat No. B661101-0001 [Sangon Biotech (Shanghai) Co., Ltd.]
