# Supplementary material for: A novel gene-expression-signature-based model for prediction of response to Tripterysium glycosides tablet for rheumatoid arthritis patients
Source: J Transl Med. 2018 Jul 4;16:187. doi: 10.1186/s12967-018-1549-9 (PMC6032531; doi:10.1186/s12967-018-1549-9)
Supplement: Supplementary file 3 — Additional file 3. Differentially expressed genes between responder and non-responder groups. [file 12967_2018_1549_MOESM3_ESM.pdf]

Additional file 3 Differentially expressed genes between responder and non-responder groups	
**Gene Symbol**	**style**
ABRA	up
ACER2	down
ACTL6B	down
AGAP11	down
ALG3	down
ANKRD16	down
ANKRD30BL	down
ANKRD45	down
BMP5	down
C11orf36	up
C12orf60	down
C1orf51	down
C21orf7	down
C2orf69	up
C3orf37	up
C4orf45	down
C6orf203	up
C7orf53	down
C8orf31	up
CALML6	down
CAV2	down
CBWD7	up
CCDC121	up
CCDC87	down
CCDC89	up
CCL19	down
CCL4L2	up
CD22	up
CD8B	down
CETP	down
CIB2	down
CRK	up
CSAG1	down
CXCL6	down
DOLPP1	down
EIF2AK4	up
ERI1	up
ERVW-1	down
ESAM	down
FAM110B	up

FAM115A	up
FAM127A	up
FAM18B2	up
FAM216B	up
FAM47A	up
FAM69B	down
FAM86C1	up
FBXO28	up
FBXW10	down
FEM1A	up
FEZ1	up
FFAR3	down
FGF5	down
FN1	up
FNDC9	up
FOXA3	up
FUT1	down
GABRR2	down
GBAP1	up
GDPD3	up
GH2	up
GHR	up
GIP	up
GJB7	up
GOLGA6L1	down
GOLGA6L5	up
GPR83	down
GRAPL	up
GRM4	down
GSTZ1	down
HAND1	up
HLA-DRB1	down
HNF1A-AS1	up
HSPB8	up
HTR3B	up
HTR3C	down
ICT1	down
ID2	up
IFI35	down
IGF1	down
IPW	down
JAZF1	up
KRTAP19-7	down

KRTAP4-12	down
LBP	down
LDLRAP1	down
LINC00304	down
LINC00474	down
LOC100131373	down
LOC100506123	down
LOC100653008	down
LOC439911	up
LOC440173	down
MAGEA10	down
MC3R	up
MDP1	up
MED18	down
MEMO1	down
MFSD9	down
MGC32805	down
MIR137HG	up
MLF1IP	up
MLLT11	down
MLN	down
MORN4	up
MRGPRX1	down
MX1	down
NAP1L2	up
NECAB3	down
NGFRAP1	down
NIT1	down
NOC2L	down
NPFFR2	down
NS3BP	up
OASL	down
OAZ1	down
OR13C9	down
OR1B1	down
OR1G1	up
OR3A2	down
OR4D9	down
OR51M1	up
OR52N5	down
OR5AU1	down
OR5B12	up
OR5P3	up

OR6C2	down
OR7E5P	up
OR7G3	up
OR8U1	up
OR9I2P	up
P4HA2	down
PBX2	up
PCDHB7	down
PDCL	up
PDGFRL	down
PLA2G10	down
PLEKHG4B	down
PMCHL2	down
PNMA2	down
PNMA6C	up
PNMT	up
PP13	down
PRH2	down
PRRG3	down
PTGFRN	down
PXT1	down
RAB28	up
RAB33B	up
RAB39B	up
RACGAP1P	up
RETSAT	up
RHEB	up
RHOXF1	up
RNASEH1	up
RNF2	up
RNF8	down
RNPS1	up
RPL23	down
RPL36AL	down
RPS2P32	down
RRP7A	up
RUSC1-AS1	up
RXRG	down
SATB2-AS1	down
SIGLEC11	up
SLC22A5	down
SLC35E3	up
SLC37A4	down

SLC45A4	up
SLC7A2	down
SMCP	down
SNX20	up
SON	up
SORL1	up
SOSTDC1	down
SPATA3	down
SPINK1	down
SPOCD1	down
SSX6	up
SUCLG1	down
TAAR1	down
TAF13	up
TAS2R20	down
TAS2R31	up
TBC1D3P2	up
TCTN2	up
TIMM23	down
TMC7	up
TMEM154	up
TMEM69	down
TNFSF18	down
TPGS2	down
TPM3P9	up
TUBA1B	down
UST	up
VAV2	up
VSTM4	up
WFDC6	up
YPEL1	up
ZNF132	up
ZNF169	up
ZNF200	up
ZNF35	up
ZNF384	up
ZNF555	up
ZNF571	up
ZNF660	down
ZNF670	down
ZNF721	up
ZNF79	up
ZSCAN10	up
